# Supplementary material for: A nonparametric mixed exponentially weighted moving average-moving average control chart with an application to gas turbines
Source: PLoS One. 2024 Aug 13;19(8):e0307559. doi: 10.1371/journal.pone.0307559 (PMC11321579; doi:10.1371/journal.pone.0307559)
Supplement: Attachment — Submitted filename: Response to Reviewer.docx [file pone.0307559.s001.docx]

**Response Letter**

**Manuscript ID:** PONE-D-24-18713

Manuscript Title: A Nonparametric Mixed Exponentially Weighted Moving Average-Moving Average Control Chart with an Application to Gas Turbines

Respected Editor and Reviewers,

Thank you very much for your valuable suggestions for the betterment of the manuscript. Following is the itemize response to the comments/suggestions.

Reviewer 1

Comment 1: The abstract should be re-written in more concise way including objective, methodology and findings.

Response: *We have revised the manuscript as suggested.*

Comment 2: The manuscript is very lengthy. To increase the readability, reduce the length of manuscript if possible.

Response: *We have incorporated the suggestion in the revised manuscript by reducing the interpretations of the results.*

Comment 3: All abbreviation should be defined as and when first time used.

Response: *The suggestion is incorporated in the revised manuscript.*

Comment 4: The experimental environment should be included in text.

Response: *The suggestion is incorporated in the revised manuscript.*

Reviewer 2

Comment 1: In the algorithm to obtain the run length profiles: It seems that steps 2 and 7 are contradictory. You have already set the parameters for a fixed value of ARL0, so why do you check if the desired ARL0 is attained?

Response: *Initially, We find the value of limit coefficient* $\left( \boldsymbol{L} \right)$ *for fixed values of other design parameters* $\boldsymbol{\eta}$ *and* $\boldsymbol{w}$ *in order to achieve the desired* $\boldsymbol{ARL}_{\boldsymbol{0}}$*. If the desired* $\boldsymbol{ARL}_{\boldsymbol{0}}$ *is not attained at that particular value of* $\boldsymbol{L}$*, we revise the value of* $\boldsymbol{L}$ *to attain desired* $\boldsymbol{ARL}_{\boldsymbol{0}}$*. This procedure is repeated until we get the desired* $\boldsymbol{ARL}_{\boldsymbol{0}}$*. After that, we have obtained the IC and OOC run-length characteristics using the finalized value of* $\boldsymbol{L}$*.*

Comment 2: Include the recently published paper [2] in Introduction Section, where the authors provided a comprehensive simulation study of zero state and steady state run-length properties of mixed control charts.

Response: *The suggested paper has been cited in the revised manuscript.*

Comment 3: Below Table 8, in the comparison of different charts, why were these chart parameters chosen? Please clarify this.

Response*: For a rational comparison between the existing and proposed control charts in Table 8, we choose those values of design parameters of the existing and proposed control charts for which* $\boldsymbol{ARL}_{\boldsymbol{0}}\boldsymbol{\cong}\boldsymbol{370}$*. In Table 8 we compare the performance of the proposed control chart with the existing control charts on the basis of shift in process proportion. Whereas, in Table 9, we compare the performance of existing and proposed control charts on the basis of various symmetric and asymmetric distributions under various parameter(s) settings.*

Comment 4: This paper focuses on scenarios where the normality assumption is not valid. However, in practice, the assumption of independence might also be invalid. The authors should, clarify this issue in revision.

Response: *This proposed control chart initially assumes that the observations must be i.i.d. (independently and identically distributed), which is the basic assumption of the sign test as described by Yang et al. (2011) and many other authors who have utilized the sign or arc sign test in their charting structure. So, our charting structure is confined to unknown or non-normal but i.i.d. observations, as already mentioned in Section 2.*

Comment 5: The manuscript is generally readable, but there are few typos present. It should undergo careful proofreading. Additionally, expanded form of abbreviations used in the manuscript must be provided when they are first introduced for clarity and comprehension.

Response: *The suggestion is incorporated in the revised manuscript.*

Yang, S.-F., Lin, J.-S. & Cheng, S. W. A new nonparametric EWMA sign control chart. Expert. Syst. Appl. 38(5), 6239-6243 (2011).
